# A benchmark survey of the common plants of South Northumberland and Durham, United Kingdom

**DOI:** 10.3897/BDJ.3.e7318

**Published:** 2015-12-29

**Authors:** Quentin J. Groom, John Liam Durkin, John O'Reilly, Andy Mclay, A John Richards, Janet Angel, Angela Horsley, Megs Rogers, Gordon Young

**Affiliations:** ‡ Agentschap Plantentuin Meise, Meise, Belgium; § John Durkin Ecology, Blaydon, United Kingdom; | Ptyxis Ecology, Lambley, United Kingdom; ¶ Botanical Society of Britain and Ireland, Hexham, United Kingdom; # The Natural History Society of Northumbria, Newcastle-upon-tyne, United Kingdom

**Keywords:** randomised survey, vascular plants

## Abstract

**Background:**

It is obvious to anyone studying plants in the landscape that man-made environmental change is having profound effects on the abundance, distribution and composition of plant communities. Nevertheless, quantifying these changes and estimating the impact of the different drivers of change is extremely difficult. Botanical surveying can potentially provide insights to the changes that are occurring and inform decisions related to conservation, agriculture and forestry policy. However, much of botanical surveying is conducted in such a way that it is not comparable between dates and places. Any comparison of historical and modern data has to account for biases in the recording of different taxonomic groups, geographic biases and varying surveying effort in time. In 2010 botanical recorders in the Vice Counties of Durham and South Northumberland in the United Kingdom decided to conduct a four year survey specifically to benchmark the abundance and distribution of common plants in their counties. It is intended that this survey will provide a relatively unbiased assessment with which to compare future and past surveys of the area and a means to study the drivers of biodiversity change in the North-east of England.

**New information:**

This survey of Durham and South Northumberland has been designed with two goals, firstly to provide information on common vascular plant species and secondly to provide a dataset that will be versatile with respect to the sorts of questions that can be answered with the data. The survey is primarily an occupancy study of 1km^2^ grid squares, however, observers were also asked to provide a relative abundance estimate of the species in each grid square. The collection of relative abundance estimate data was an experiment to assess the repeatablity and useablity of such estimates.

## Introduction

There is a need for active monitoring of organisms and habitats in the wild, not just for curiosity, but to inform us of the changes that are occurring. Environmental change is often reported anecdotally and causation is assumed, but without at least semi-quantitative measurements we cannot hope to unravel the complex interacting factors that are really driving changes. Data are needed to inform decision makers on all aspects of management that affect the countryside, including conservation, land management and farming.

The North-east of England is fairly typical of the landscapes found in the rest of the United Kingdom. It has large urban areas, a long coastline, large expanses of arable land, extensive grazing land, forestry and upland moorland. For biological recording purposes Great Britain and Ireland are divided into Vice Counties, which have permanent borders. The Botanical Society of Britain and Ireland appoints voluntary Vice County Recorders (VCR) to each Vice County and this survey is the result of a collaboration between the VCRs of Durham (JD) and South Northumberland (AJR, QG). The region has a number of active amateur biological recorders and this survey was also seen as a means to give direction to their recording effort.

There are many factors driving biodiversity change in the North-east England, most are common to other areas of Northern Europe, whereas others are more local. Below are listed some of these drivers that could be explored further using these data.

Eutrophication from agricultural fertilizers, waste and atmospheric deposition has become an insidious and pervasive driver of habitat change (Duprè et al. 2010, Phoenix et al. 2006, Stevens et al. 2004). Not only does eutrophication impact places where there is direct application of fertilizer, such as on farmland, but also isolated wild areas are affected through atmospheric deposition. Atmospheric nitrogen deposition is also a cause of soil acidification, to which sulphur emissions also contribute, though the latter have declined in recent years.

A particular land use change to the North-eastern England has been the conversion of peat moorland into conifer plantations. A notable example in South Northumberland is Kielder Forest, the largest man-made forest in England, it covers 60,000 hectares in the west of the county along the Scottish and Cumbrian border (Forestry Commission, England 2006). About three-quarters of the plantation is Sitka spruce (*Picea sitchensis* (Bong.) Carr) and there are also large plantations of Norway spruce (*Picea abies* (L.) Karsten) and Scots Pine (*Pinus sylvestris* L.).

Artificial drainage has also been the cause of significant habitat change. A notable historic example was the drainage of Prestwick Carr in the 19^th^ century that led to the local extinction of many species (Groom et al. 2014). The few remaining lowland wetlands are now largely protected from drainage. However, drainage of the uplands is still continuing in order to extend conifer plantations.

Farming practises directly and indirectly change habitats and the landscape. The mechanization of farming occurred some time ago, as did the introduction of modern herbicides and pesticides; however, agriculture continues to change with the introduction of new crops, the changing profitability of livestock versus arable farming and new policies intended to promote good stewardship of the countryside (Robinson and Sutherland 2002, Storkey et al. 2011). Even on non-agricultral land the use of amenity seed mixes to vegetate large areas has changed natural vegetation and introduced non-native taxa and novel genotypes of native species.

Urbanisation, industrialisation and associated development have profoundly changed the environment locally within the region, especially in the eastern lowlands. Smaller-scale developments are ongoing, but mostly confined to areas that have already been developed. Mining of various minerals was a particularly important economic activity in this region and few parts of the region were unaffected by it. Following the relatively recent decline of the coal industry, many former coal mine sites were landscaped and ‘tidied up’. In more recent times mining and quarrying activities have been more localised. Opportunities for wild plants to colonise and survive have been dramatically altered by all of these activities.

Alien species may also have an impact on native communities, though it is difficult to separate their influence from other habitat change. Alien plants exert competitive pressure, but there are also emerging diseases and introduced animals that may exert a pathogenic or herbivorous pressure. The distribution ranges of insects have been moving north in recent years, presumably as a consequence of climate change (Hill et al. 2011). Climate change may ultimately have the greatest impact on the diversity and distribution of plants, but so far its impact on plant distributions is not yet clear above the signal of other environmental change (Groom 2013).

Observations of wild plants in the counties have been made at a number of different spatial resolutions, 1km2, 4km2, 25km2 and 100km2. However, the trend in recent years has been towards finer resolutions. This has been driven by the availability of digital systems for storing observations and by access to systems for mapping and analysing the data. The current survey used a grid of 1km2 and although even finer resolutions would give greater sensitivity to change, 1km2 grid squares are advantageous from many perspectives. This resolution is close to the scale of many landscape features in the English countryside, fields, towns, lakes and hills. They allow surveyors to cover a large area in a reasonable amount of time. This grid square is also clearly indicated on Ordnance Survey maps and on Global Positioning Systems.

From a policy perspective much emphasis is placed on the conservation of rare species even though common plants are those that are most important for ecosystem health and function. The focus of this survey is on those common species and their habitats.

From a statistical perspective there are a large number of options for distributing survey sites. For example, stratification can be used to ensure even representation of different habitat types. Sites can also be distributed non-randomly to evenly cover the environmental space of an area and reduce the impact of spatial autocorrelation. Nevertheless, a completely random approach was chosen to make the results as versatile as possible for whichever questions may in the future be resolved using these data. Some types of analyses may have reduced statistical power when used with a completely random design, but a random survey avoids having to make assumptions about the drivers of changes that may occur in the future and their location.

This approach is not strongly hypothesis driven. However, this is not necessarily a disadvantage. To some extent hypothesis driven monitoring is likely to produce more robust results than undirected monitoring. However, it can also be argued that general monitoring has the advantage of detecting unexpected changes that targeted monitoring would miss (Wintle et al. 2010). We have seen, and expect to see further environmental change in this region. Some of those future changes are already known, such as climate change. However, the stochastic nature of the environment and the unpredictability of human activities mean that accurate forecasting is impossible. The challenge in the future will be to use these data to identify real change before it becomes readily apparent and use the results to adapt policy in a positive way.

### Former botanical surveys of the North-East of England

The first observations of plants in North-East England come from William Turner [ca. 1508 – 1568] (Raven 1947). However the first systematic floras of the region were written by Nathaniel John Winch [1768 – 1838] (Winch 1805, Winch 2014), followed by John Gilbert Baker [1834 – 1920] and George Tate [1805 – 1871] (Baker and Tate 1868). Throughout the 19^th^ and 20^th^ centuries several societies contributed to our knowledge on the flora, these include The Natural History Society of Northumbria, the Cleveland Naturalists' Field Club and the Northern Naturalists' Union. Through their activities and their publications few species can have gone unnoticed in the region. The most recent flora for Durham was published in 1988 by the Reverend George Gordon Graham [1917 – 2015] and contains detailed species accounts and maps. It is based upon a survey of 4km^2^ grid squares in the county between 1968 and 1988 (Graham 1988). In South Northumberland the most recent flora was published in 1993 by Professor George Albert Swan [1917 – 2012], it is based upon observations collected from 1968 onward, using a 25km^2^ grid system (Swan 1993). In 2001 supplements to both county floras were published with additional records and corrigenda (Swan 2001, Graham 2001​). In recent years both counties have published Rare Plant Registers, which catalogue the rare and scarce plants of the counties, detailing the remaining sites and the conservation status of the species at these sites (Durkin 2014, Groom et al. 2014). Digitization of the historic records began around the turn of the millennium and is still continuing. Almost 400,000 paper-based records have so far been digitised.

Since 2007 all available computerised botanical records for the region have been displayed publically on distribution maps through the Flora of North-East England website (Groom 2015). The records displayed on these web-based distribution maps are significantly more comprehensive and up-to-date than either of the published Floras and are updated regularly.

## Project description

### Title

The North-East Common Plants Survey

### Personnel

All personnel on this survey were volunteers and had a range of experience in plant identification and botanical surveying. Some were either professional or retired biologists and ecologists, while others are amateurs, though their experience ranged from expert to beginner. The vast majority of observations were made by the more experienced contributors. More than 70 people contributed to the data collected for the project, but the majority of surveys were conducted by the authors, either as individuals or as groups. Conduct and safety advice was provided to the volunteers with links to the standard advice given by the BSBI (Palmer and Hearn 1999, Rich 2000).

### Study area description

The Watsonian Vice Couties of Durham and South Northumberland cover an area of 6134 km^2^. Durham’s highest point is Mickle Fell (788m) and South Northumberland’s is Kilhope Law (673m). These counties contain a wide variety of natural and man-made habitats, though those most relevant to this survey are the most extensive. These are upland moors, grazing pasture, arable farming, plantation forestry and urban areas. Other scattered, but common habitats are deciduous woodland, sphagnum bog and freshwater. The area also includes large parts of the North Pennines Area of Outstanding Natural Beauty and Northumberland National Park.

### Design description

Surveys were conducted using the one kilometre grid squares of the Ordnance Survey (Datum: OSGB36; EPSG:27700). Two hundred 1km^2^ squares were chosen randomly from all squares in the two Vice Counties, except for squares that fell within the Otterburn Army Training Estate in the north-west of South Northumberland. Random numbers were generated using Microsoft Excel. Only squares with at least 50% of their land within either Durham or South Northumberland were included, though all randomly chosen grid squares with a proportion of open water were included in the survey. Only one square had more than 50% of its area covered by sea. Seven of the randomly selected squares had no public access and were substituted. To avoid spatial bias the substitute squares were randomly selected from one of the four adjoining squares.

Public access to the countryside in the area is quite extensive. Not only were there public footpaths, permissive footpaths, bridleways and common land, but in the west of the counties there are extensive areas of Open Access Land which can be walked freely. In some cases permission was obtained to visit particular sites, specifically some of the lakes that were contained within the survey area. None of the selected squares had areas that were physically impossible to visit, though some in the west are several kilometres from the nearest paved road.

A website created for the survey indicated to volunteers where surveys had already been conducted and was updated regularly. Squares were shown on a map to indicate whether the grid square had already been surveyed in spring, summer, surveyed twice or surveyed three or more times. When requested, suggestions were made to surveyors to guide them where to go. However, there was no attempt to allocate areas to particular surveyors or insist that surveyors should visit particular squares. It was suggested to surveyors that conducting two surveys in different seasons per grid square would be ideal. In the final year the first author made a particular effort to complete squares that had not been covered in the previous surveys.

### Funding

This survey has been conducted without external funding.

## Sampling methods

### Sampling description

The surveyors were asked to visit the full range of habitats within the grid square and to look over the whole area. After completing the survey they were asked to assign a DAFOR score (Dominant, Abundant, Frequent, Occasional, Rare) to the relative abundance of the species within the grid square. As there were many ways that the surveyors could interpret the DAFOR scores, written guidelines were also provided (Suppl. materials 1, 2). Surveys were recorded on paper, mostly on recording cards that were provided (Suppl. materials 3, 4, 5, 6). Some grid squares spanned Vice County boundaries, particularly along rivers Coquet, Tyne and Derwent and on the watershed. In these squares surveyors were asked to record full lists on two separate cards in these squares, one card for each vice-county.

The vascular plant biodiversity and landscape complexity varied considerably between sample squares. For this reason there was no attempt to balance the recording time between squares. Heterogeneous areas with a mosaic of habitats in the lowlands required more effort than comparatively uniform areas in the hills. It was left to the individual surveyors to determine when they had completed their survey. However, in the final year additional surveys were conducted in some grid squares deemed to be insufficiently surveyed.

The numbers of surveys conducted for each grid square are summarised in Table 1. DAFOR scores were not recorded for every survey. The two main reasons for surveyors not assessing DAFOR scores were that either the whole square had not been surveyed, or the surveyor was unaware of the requirement to do the assessment.

Although the goal was to survey all 200 selected squares over the four years of the survey, 35 were not surveyed and a further two were surveyed, but incompletely (Fig. 2). 339 surveys were conducted on the remaining 163 squares and 160 had at least one survey were DAFOR estimates were provided (Table 1).

### Quality control

All records were reviewed by Quentin Groom and John Durkin upon arrival and questionable records were queried with the observer as soon as possible after receiving the observations. All data where entered into the database system Mapmate (Mapmate Ltd., UK). This data entry system validates the data upon entry, warning the user of potential incorrect dates, exceptional species and malformed or misplaced grid references. The Mapmate database also hold most of the historic observations of Northumberland's and Durham's flora and allows these to be mapped. Visualization of the distributions of observations was another tool used to locate potential errors. For taxa that are particularly difficult to identify specimens were sent to the BSBI's panel of referees and specialists. Determination details are provided with the records.

## Geographic coverage

### Description

The survey covered the Watsonian Vice Counties of Durham and South Northumberland in north-east England. The boundary of Durham follows the course of the River Tees to the south and the Rivers Tyne and Derwent to the north where it borders South Northumberland. The boundary of South Northumberland follows the River Coquet to the north, but has a less distinct boundary to the west. It largely follows the Pennine ridge along the border with Scotland and Cumberland, but in a section it also follows the River Irthing, a tributary of the River Eden.

### Coordinates

54.450713 and 55.368047 Latitude; -2.690092 and -1.153764 Longitude.

## Taxonomic coverage

### Description

The survey covered all vascular plants and Characeae growing in the wild, whether native or alien. The taxonomy of Vascular plants follows Stace 2010. The taxonomy of the Characeae follows John et al. 2011

## Temporal coverage

**Data range:** 2010 1 01 – 2013 12 31.

### Notes

**Figure 1.**
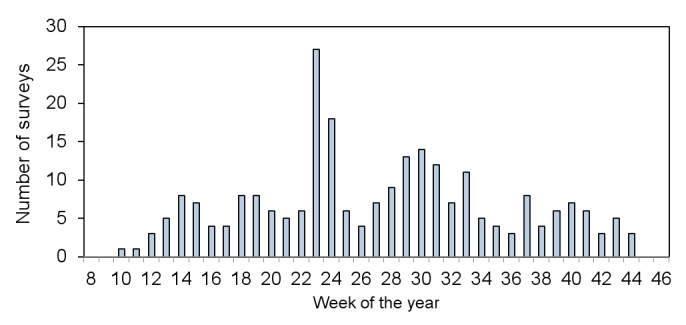
The temporal distribution of surveys over the four years (2010 – 2013) of the project, pooled by the week of the year that each survey was conducted.

**Figure 2.**
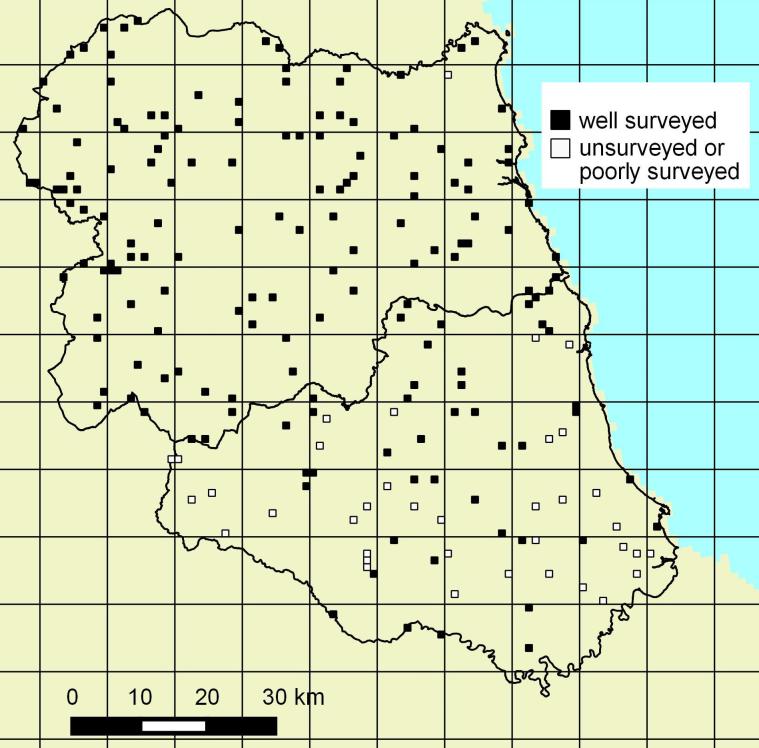
The distribution of randomly selected grid squares in Durham and South Northumberland. Selected sites that remained unsurveyed or have been inadequately surveyed over the four years are indicated. The vice county boundary data is public sector information licensed under the Open Government Licence v3.0.

The detectability and identifiability of many species varies with the season. For this reason there was a conscious effort to survey areas more than once in different seasons. This is particular relevant to lowland areas and woodland, where spring ephemerals and agricultural weeds are only visible for a short season. Fig. 1 shows the temporal distribution of surveys over the four years of the project. Surveys can be seen to be well-distributed over the whole season peaking in the main summer season, but broadly distributed.

## Usage rights

### Use license

Creative Commons CCZero

### IP rights notes

These data have been made available in the public domain with the hope that they will be used to improve our knowledge on the British flora. However, we expect that users of these data will conform to the normal conventions of scientific citation.

## Data resources

### Data package title

A common plants survey of vascular plants in South Northumberland and Durham, United Kingdom

### Resource link

http://www.gbif.org/dataset/5d784d06-fa1d-4f00-8cdc-663d04d26061

### Alternative identifiers

doi:10.15468/qodsto

### Number of data sets

1

### Data set 1.

#### Data set name

A common plants survey of vascular plants in South Northumberland and Durham, United Kingdom

#### Data format

DWC-A

#### Character set

utf-8

#### Download URL

http://apm-ipt.br.fgov.be:8080/ipt-2.3.2/archive.do?r=commonplantssurveyofvascularplantsnortheastengland

#### Data format version

1.4

#### Description

The data source contains all survey details from the period of the survey 2010 – 2013. However, it also includes miscellaneous observations back to 1998. These additional observations which may be used to fill gaps where they exist in the surveying effort.

## Additional information

### Suggested use of the data

The unsurveyed grid squares are at odds with the goal of having an unbiased dataset that covers the two counties. South Northumberland was almost completely surveyed, but County Durham was incompletely surveyed with unsurveyed squares concentrated particularly in the south. There was no obvious prejudice of recorders against particular habitats; however, it appears that these unsurveyed squares are unsurveyed because they are distant from the homes of active surveyors.

Apart from ignoring these missing data, users of these data could resolve this problem in at least two ways. Analysis could be conducted only on the well-surveyed portion of the area, or surveys conducted before 2010 or after 2013 could be used to fill gaps where these observations exist. Eleven of the unsurveyed squares had surveys from between 1998 and 2009 and these surveys have been included in the dataset.

To demonstrate a potential use of these data, universal kriging has been used to interpolate DAFOR scores for *Calluna vulgaris* (Fig. 3a). The associated kriging variances conveniently demonstrate where there are spatial information gaps (Fig. 3b). This map clearly shows the large degree of uncertainty in southern Durham. Another gap is in the north-west of the area where the Otterburn Firing Ranges prevented access. There is also an obvious edge effect to the interpolation where, owing to the random distribution of the sites, locations at the edge of the region are supported by fewer neighbouring sampled sites.

## Supplementary Material

Supplementary material 1 Guidance notes for recording DAFOR scores Data type: text
Brief description: To harmonize the approach of recorders to the assignment of DAFOR abandance scores guidance notes were provided. This document contains those original guidance notes.
File: oo_43350.pdf O'Reilly, John

Supplementary material 2 Text version of the guidance notes for recording DAFOR scores Data type: text
Brief description: To harmonize the approach of recorders to the assignment of DAFOR abandance scores guidance notes were provided. This document contains those original guidance notes.
File: oo_71136.txt O'Reilly, John

Supplementary material 3 Recording card for Durham Data type: text
Brief description: The recording card for Durham provided for surveyors to collect their observations on. The card uses abbreviated Latin names for the most common plants of the area and BRC Code numbers created by the Biological Records Centre, these numbers speed data entry.
File: oo_43352.pdf Groom, Quentin

Supplementary material 4 Recording card for South Northumberland Data type: text
Brief description: The recording card for South Northumberland provided for surveyors to collect their observations on. The card uses abbreviated Latin names for the most common plants of the area and BRC Code numbers created by the Biological Records Centre, these numbers speed data entry.
File: oo_43353.pdf Groom, Quentin

Supplementary material 5 An XSL-FO version of the recording card for Durham Data type: XML
Brief description: The XSL-FO version of the Durham recording card that can be processed with Apache FOP to recreate the PDF version. It is included to allow the creation of edited versions of the card.
File: oo_71137.fo Groom, Quentin

Supplementary material 6 An XSL-FO version of the recording card for South Northumberland Data type: XML
Brief description: The XSL-FO version of the South Northumberland recording card that can be processed with Apache FOP to recreate the PDF version. It is included to allow the creation of edited versions of the card.
File: oo_71138.fo Groom, Quentin

## Figures and Tables

**Figure 3a.**
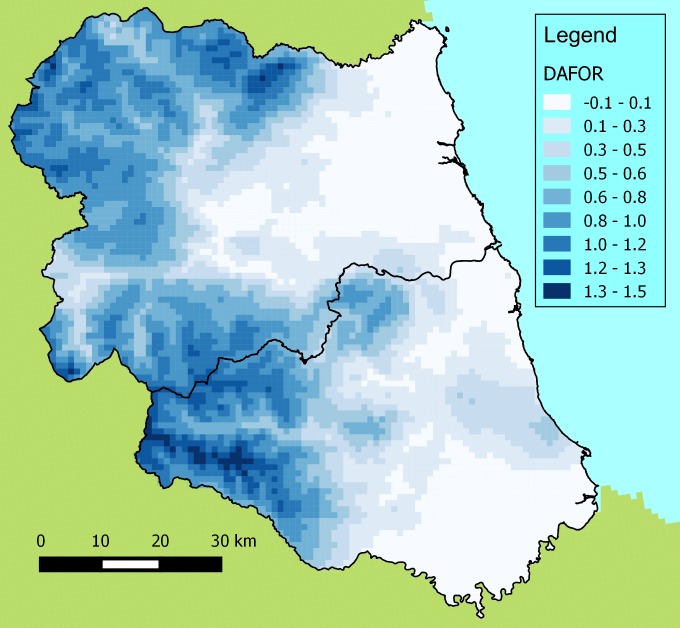
The interpolated abundance estimated from DAFOR scores of *Calluna vulgaris* from all surveys. Interpolation was conducted using universal kriging with altitude used as the covariable. The variogram was constructed using a width of 2,000m and a cutoff of 40,000m.

**Figure 3b.**
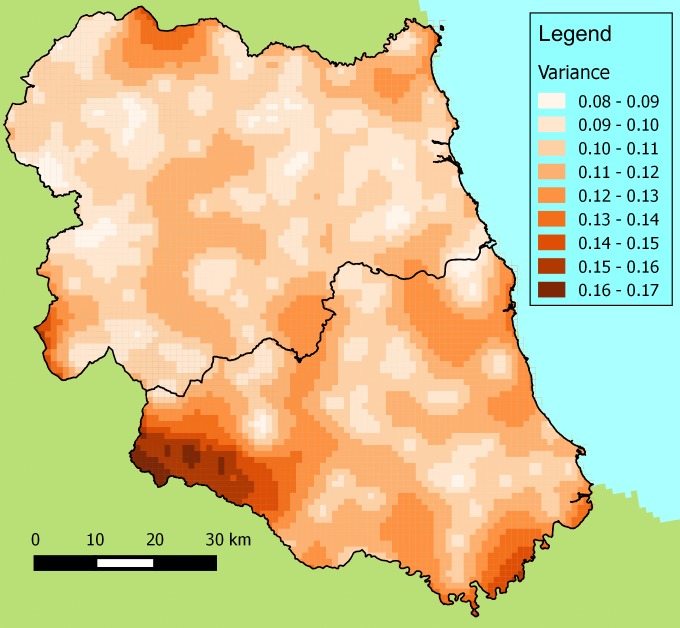
The kriging variances of the interpolated DAFOR scores.

**Table 1.** A summary of the surveyed grid squares and the numbers of visits to them. The numbers of surveys are separated by whether each species was assigned a DAFOR abundance estimate. Each square was assessed as to whether it had been well surveyed. This assessment is based on the number and timing of surveys and on the diversity of habitats within the grid square. It is a rough guide to users of these data as to the intensity of surveying at each site.

**Grid Reference**	**Site Name**	**Vice County**	**well surveyed**	**with DAFOR scores**	**without DAFOR scores**
NT6401	Green Needle Burn	South Northumberland	yes	1	0
NT6602	Carry Burn	South Northumberland	yes	1	0
NT6905	Black Cleugh	South Northumberland	yes	1	0
NT7001	Girdle Fell	South Northumberland	yes	1	0
NT7205	Lumsdon Law	South Northumberland	yes	2	0
NT7406	Catcleugh Hill	South Northumberland	yes	1	0
NT9303	Harbottle Wood	South Northumberland	yes	2	1
NT9502	Holystone	South Northumberland	yes	1	1
NU2202	Calvil Head	South Northumberland	yes	1	0
NU2403	North Togston	South Northumberland	yes	2	0
NY5790	Bloody Bush	South Northumberland	yes	1	0
NY5882	Black Knowe	South Northumberland	yes	1	0
NY5982	Dinmont Lairs	South Northumberland	yes	1	0
NY6097	Deadwater Rigg	South Northumberland	yes	1	1
NY6281	Between Slighty Crags and Black Knowe	South Northumberland	yes	2	0
NY6293	Kielder village	South Northumberland	yes	2	0
NY6368	Wardrew Wood	South Northumberland	yes	1	2
NY6381	West of Black Knowe	South Northumberland	yes	2	0
NY6479	Reamy Rigg	South Northumberland	yes	2	0
NY6483	Humble Burn	South Northumberland	yes	2	0
NY6581	East of Black Knowe	South Northumberland	yes	1	0
NY6588	north-east of Leaplish	South Northumberland	yes	1	2
NY6670	Peat Rigg	South Northumberland	yes	1	0
NY6678	Hurtle Winter	South Northumberland	yes	2	0
NY6849	Dearquarry Sike	South Northumberland	yes	2	1
NY6859	Coanwood	South Northumberland	yes	3	0
NY6862	Wydon Eals	South Northumberland	yes	3	1
NY6951	Slaggyford, near.	South Northumberland	yes	1	0
NY6969	West of Whiteside	South Northumberland	yes	1	0
NY6977	Little Gowany Knowe	South Northumberland	yes	2	0
NY7069	Whiteside	South Northumberland	yes	2	0
NY7070	Burndivot Common	South Northumberland	yes	1	0
NY7084	Dings Rigg	South Northumberland	yes	2	0
NY7097	Smallhope Sikes	South Northumberland	yes	2	0
NY7169	Brown Rigg	South Northumberland	yes	1	0
NY7191	Hawkhope Burn	South Northumberland	yes	2	0
NY7290	The Cross	South Northumberland	yes	2	0
NY7350	Ayle Burn	South Northumberland	yes	2	0
NY7364	High Town	South Northumberland	yes	2	0
NY7371	Hopealone	South Northumberland	yes	2	1
NY7373	Jock's Close Hill	South Northumberland	yes	2	1
NY7455	Blaeberry Cleugh	South Northumberland	yes	1	0
NY7548	Carrier's Hill	South Northumberland	yes	2	0
NY7571	Drove Rigg	South Northumberland	yes	1	0
NY7685	The Eals	South Northumberland	yes	2	0
NY7692	Coals Cleugh	South Northumberland	yes	1	0
NY7760	Kingswood Burn	South Northumberland	yes	3	0
NY7776	White Hill	South Northumberland	yes	2	0
NY7787	Thorneyburn Common	South Northumberland	yes	3	0
NY7853	Ninebanks	South Northumberland	yes	1	1
NY7866	Thorngrafton	South Northumberland	yes	3	2
NY7889	Heathery Hall	South Northumberland	yes	3	0
NY7892	Ridley Shiel	South Northumberland	yes	2	0
NY7941	Nag's Head	Durham	no	0	0
NY7982	Mesling Crags	South Northumberland	yes	1	0
NY8041	Wellhope Moor	Durham	no	0	0
NY8054	Round Hill	South Northumberland	yes	1	1
NY8071	Folly Lake	South Northumberland	yes	1	1
NY8090	Burdonside	South Northumberland	yes	1	1
NY8235	Grasshill Common	Durham	no	0	0
NY8244	Middlehope Moor	Durham & South Northumberland	yes	1	2
NY8285	Sheel Law	South Northumberland	yes	1	0
NY8395	Kellyburn Hill	South Northumberland	yes	1	0
NY8444	Westend Moor	South Northumberland	yes	1	0
NY8451	Sinderhope	South Northumberland	yes	2	2
NY8536	Noon Hill	Durham	no	0	0
NY8730	Wool Pits Hill	Durham	no	0	0
NY8848	Halleywell Fell	South Northumberland	yes	1	0
NY8850	Nevin Sike	South Northumberland	yes	1	0
NY8885	Cragg Farm	South Northumberland	yes	1	0
NY8963	Low Gate (west of)	South Northumberland	yes	1	1
NY8975	Short Moor	South Northumberland	yes	2	0
NY8991	Silvernut Well	South Northumberland	yes	2	0
NY8994	Fawdon Hill	South Northumberland	yes	1	0
NY9161	West Dipton Burn	South Northumberland	yes	3	2
NY9165	West Boat to A69 bridge	South Northumberland	yes	2	2
NY9433	Out Berry Plain	Durham	no	0	0
NY9465	West Oakwood area	South Northumberland	yes	1	1
NY9577	Carrier's Lane	South Northumberland	yes	2	0
NY9646	Far Sandy Ford	Durham	yes	1	0
NY9659	Woolley Hospital	South Northumberland	yes	2	1
NY9689	Todcrag Moss	South Northumberland	yes	1	1
NY9697	Darden Burn	South Northumberland	yes	1	0
NY9699	Harehaugh Hill	South Northumberland	yes	2	0
NY9754	Winnows Hill	South Northumberland	yes	2	1
NY9875	Hallington	South Northumberland	yes	1	2
NY9889	East of Birky Burn	South Northumberland	yes	1	0
NY9937	Thimbleby Hill	Durham	yes	1	1
NY9939	Stanhope	Durham	yes	0	3
NZ0039	Jollybody Farm	Durham	yes	3	1
NZ0048	Harehope Lead Mines	Durham	yes	2	1
NZ0050	Edmondbyers Common	Durham	yes	1	0
NZ0143	Waskerley Park	Durham	no	0	0
NZ0162	Styford Hall	South Northumberland	yes	2	2
NZ0181	Kidlaw	South Northumberland	yes	2	0
NZ0189	Harwood Gate	South Northumberland	yes	2	0
NZ0192	West of Greenleighton	South Northumberland	yes	2	0
NZ0247	Cross Rig	Durham	no	0	0
NZ0318	Tees Bank	Durham	yes	1	1
NZ0369	North of Wall Houses	South Northumberland	yes	2	0
NZ0377	The Tofts	South Northumberland	yes	1	0
NZ0481	West Shaftoe	South Northumberland	yes	1	0
NZ0492	Ewesley Fell	South Northumberland	yes	1	0
NZ0497	Spylaw	South Northumberland	yes	2	0
NZ0582	Shaftoe Crags	South Northumberland	yes	2	1
NZ0599	Garleigh Moor	South Northumberland	yes	4	0
NZ0632	South-west of Doctor's Gate	Durham	no	0	0
NZ0666	Bogle Burn	South Northumberland	yes	1	0
NZ0672	How Burn, Fenwick	South Northumberland	yes	1	1
NZ0683	Corridge	South Northumberland	yes	1	1
NZ0691	Ewesley Gill	South Northumberland	yes	2	0
NZ0786	Angerton Lake	South Northumberland	yes	2	0
NZ0825	Copley	Durham	no	0	0
NZ0826	Lunton Hill	Durham	no	0	0
NZ0827	Crake Scar Farm	Durham	no	0	0
NZ0834	Shull Bank	Durham	no	0	0
NZ0924	Gibbsneese Plantation	Durham	yes	1	0
NZ1137	Thornley	Durham	no	0	0
NZ1142	Broomshiels Hall	Durham	yes	2	1
NZ1229	Little Burn	Durham	yes	1	0
NZ1248	Knitsley	Durham	no	0	0
NZ1289	West of Stanton	South Northumberland	yes	2	0
NZ1362	Greenside and Fell Farm	Durham	yes	2	1
NZ1376	Cuthburt's Nook	South Northumberland	yes	1	0
NZ1398	Weldon	South Northumberland	yes	3	0
NZ1416	Winston	Durham	yes	3	2
NZ1450	Iveston	Durham	yes	1	0
NZ1464	Barmoor	Durham	yes	1	0
NZ1534	south-west of Crook	Durham	no	0	0
NZ1538	Billy Hill	Durham	yes	2	1
NZ1552	north-west of Annfield Plain	Durham	yes	1	0
NZ1570	Darras Hall	South Northumberland	yes	3	0
NZ1580	South of Shilvington	South Northumberland	yes	1	1
NZ1583	South East of Molesden	South Northumberland	yes	1	0
NZ1590	Abshiel	South Northumberland	yes	3	0
NZ1644	Click-Em-Inn Farm	Durham	yes	2	0
NZ1758	Gibside	Durham	yes	4	1
NZ1826	West Aukland	Durham	yes	1	0
NZ1838	Birk's Wood	Durham	yes	2	0
NZ1872	Prestwick	South Northumberland	yes	4	0
NZ1915	Low Field	Durham	yes	1	0
NZ1932	Hunwick	Durham	no	0	0
NZ1961	Derwent, Dam head	Durham	yes	4	1
NZ1987	Fulbeck Grange	South Northumberland	yes	3	0
NZ2027	Green Lane, Bishop Auckland	Durham	no	0	1
NZ2098	North of Eshott	South Northumberland	no	1	0
NZ2121	Houghton Grange	Durham	no	0	0
NZ2148	Charlaw Plantation	Durham	yes	2	0
NZ2171	Havannah Nature Reserve	South Northumberland	yes	2	0
NZ2182	Clifton Lane	South Northumberland	yes	2	0
NZ2252	Eden Hill farm	Durham	yes	1	0
NZ2254	Pockerley	Durham	yes	1	0
NZ2273	Big Waters Country Park west	South Northumberland	yes	2	0
NZ2373	Big Waters Country Park east	South Northumberland	yes	2	2
NZ2381	Nedderton	South Northumberland	yes	2	0
NZ2385	Paddock Hall Farm	South Northumberland	yes	2	0
NZ2435	Claxburn Wood	Durham	yes	2	0
NZ2448	Nettlesworth West	Durham	yes	2	0
NZ2477	Bassington Industrial Estate	South Northumberland	yes	2	0
NZ2830	Chilton Industrial Estate	Durham	yes	1	0
NZ2843	Kieper Farm	Durham	yes	4	0
NZ2893	West of Cresswell	South Northumberland	yes	6	1
NZ2924	High Copelaw	Durham	no	0	0
NZ2975	East Cramlington pond area	South Northumberland	yes	3	0
NZ2985	North Seaton Colliery	South Northumberland	yes	3	0
NZ2987	Summerhouse Lane	South Northumberland	yes	2	0
NZ3129	Nunstainton East	Durham	yes	1	0
NZ3143	Broomside	Durham	yes	2	0
NZ3213	Morton Park	Durham	yes	1	0
NZ3219	Moor House	Durham	yes	0	1
NZ3264	Hebburn	Durham	yes	0	1
NZ3266	Willington Quay	South Northumberland	yes	2	0
NZ3279	Blyth South Beach	South Northumberland	yes	2	0
NZ3329	Low Hardwick Farm	Durham	no	0	0
NZ3334	Garmondsway	Durham	no	0	0
NZ3359	Hylton Bridge	Durham	no	0	0
NZ3365	Jarrow	Durham	yes	2	2
NZ3461	Boldon Colliery	Durham	yes	2	0
NZ3524	Rafferdene	Durham	no	0	0
NZ3544	Hetton le Hill Wood	Durham	no	0	0
NZ3560	Boldon Golf Club	Durham	yes	2	0
NZ3566	N Sea Ferry-terminal	Durham & South Northumberland	yes	2	0
NZ3668	Low Lights	Durham & South Northumberland	yes	1	0
NZ3671	Cullercoats	South Northumberland	yes	3	0
NZ3735	Trimdon Grange	Durham	no	0	0
NZ3745	South Hetton	Durham	no	0	0
NZ3858	Southwick	Durham	no	0	0
NZ3948	Dalton Moor	Durham	yes	1	0
NZ3949	Seaton	Durham	yes	1	0
NZ4022	north-east of Carlton	Durham	no	0	0
NZ4029	Lumpley's Covert	Durham	yes	1	0
NZ4236	Hutton Henry	Durham	no	0	0
NZ4320	Durham Road, Stockton-on-Tees	Durham	no	0	0
NZ4531	Dovecote	Durham	no	0	0
NZ4628	Springwell House Farm	Durham	no	0	0
NZ4738	Green Stairs	Durham	yes	1	1
NZ4824	Cowpen Bewley	Durham	no	0	0
NZ4827	West of Greatham	Durham	no	0	0
NZ5027	Graythorp	Durham	no	0	0
NZ5131	Bellevue	Durham	yes	1	1
