# Supplementary material for: A benchmark survey of the common plants of South Northumberland and Durham, United Kingdom
Source: Biodivers Data J. 2015 Dec 29;(3):e7318. doi: 10.3897/BDJ.3.e7318 (PMC4699291; doi:10.3897/BDJ.3.e7318)
Supplement: Supplementary material 1 — Guidance notes for recording DAFOR scores [file biodiversity_data_journal-3-e7318-s001.pdf]

Guidance notes for recording DAFOR scores

In order to make the data more useful we would like you to record the relative abundance of each species you find in the square on the DAFOR scale: **D** = Dominant; **A** = Abundant, **F** = Frequent, **O** = Occasional, **R** = Rare. To do this, tick species off on your list as you find them in the normal way. When you have finished surveying the square, assign a DAFOR letter to each species.

The trick to doing this is to go through the list quickly and for each species write the first score that comes into your head. Try not to think about it too much.

If a species seems intermediate between two categories and you are unsure which to assign to it, choose the lower category, e.g. if you are unsure if something was occasional or frequent, choose occasional.

D for Dominant

In practice you will rarely, if ever use this. To score **D,** a species would have to be the most common plant by far, in well over three quarters of the square. It is possible that in a square that is entirely conifer plantation, that Sitka spruce Picea sitchensis might score **D;** or in a square that is almost all occupied by highly improved grassland, perennial rye-grass Lolium perenne might sometimes score **D**, but even these two scenarios are unlikely most of the time. If you are not sure if something should score **D** or **A**, give it **A**.

A for Abundant

Only use **A** if the plant was really very common in many parts of the square. For most species this would mean that there were thousands of individual plants present. In most squares, few species will score as highly as **A** and in quite a few squares there will be no species that score that highly. If you are not sure if something should score **A** or **F**, give it **F**.

F for Frequent

Use **F** if you found the plant in several places in the square and there was usually more than just **A** few individuals in each of these places. You could also use **F** if the plant was only present in one part of the square but was very common in that part, with many individuals and covered **A**substantial area (e.g. between one eight and one quarter of the area of the whole square). If you are not sure if something should score **F** or **O**, give it **O**.

O for Occasional

Use **O** for species that occur in several places in the square, but whose populations are usually not very big. You would also use **O** for species that are very common in one bit of habitat within the square that occupied just a small area (e.g less than one eight of the area of the whole square). You will use **O** for many species in most squares. If you are not sure if something should score **O** or **R,** give it **R**.

R for Rare

Use **R** for any species that occur as a small number of individuals in the square. This small number of individuals may be located in one place in the square, or scattered over several different locations within the square. In many squares **R** is likely to be the score that most species get. If you are not sure if something should score **O** or **R,** give it **R**.

For those of you who are used to using the DAFOR scale, please stick to the basic 5 scores only and avoid entries like **O/F** (occasional to frequent) and particularly please avoid using the prefix '**L**' as in **LF** (locally frequent).
