# Supplementary material for: A benchmark survey of the common plants of South Northumberland and Durham, United Kingdom
Source: Biodivers Data J. 2015 Dec 29;(3):e7318. doi: 10.3897/BDJ.3.e7318 (PMC4699291; doi:10.3897/BDJ.3.e7318)
Supplement: Supplementary material 3 — Recording card for Durham [file biodiversity_data_journal-3-e7318-s003.pdf]

Optimised for the Vice County of Durham (66)

| | | | | | | | | | | | | | | | | | |
|---|---|---|---|---|---|---|---|---|---|---|---|---|---|---|---|---|---|
| 3 | Acer cam | 237 | Beton off | 506 | Chrys opp | 733 | Erige acr | 687 | non | 1256 | Melam pra | 1507 | tri | 7888 | x duma | 2007 | Stell als |
| 4 | pla | 239 | Betul pen | 513 | Circa lut | 740 | Eriop ang | 999 | Hydro vul | 1263 | Melic uni | 1514 | Polyg ser | 1726 | Rubus caesi | 2009 | gra |
| 5 | pse | 240 | pub | 515 | Cirsi arv | 744 | vag | 1003 | Hyper and | 1265 | Melil alt | 1515 | vul | 3395 | das | 2010 | hol |
| 7 | Achil mil | 243 | Black per | 518 | het | 745 | Erodi*cic | 1010 | hirsut | 1267 | off | 1523 | Polyg avi | 3409 | ebor | 2012 | med |
| 9 | pta | 244 | Blech spi | 520 | pal | 4342 | Eroph ver | 1006 | mac | 1272 | Menth aqu | 1522 | *avi | 1728 | *fru | 2014 | nem |
| 19 | Adoxa mos | 1860 | Bolbo mar | 522 | vul | 753 | *ver | 1014 | per | 1285 | spi | 1544.2 | Polyp vul | 1729 | ida | 2021 | Succi pra |
| 20 | Aegop pod | 250 | Brach syl | 533 | Cochl dan | 762 | Euony eur | 1015 | pul | 1289 | Menya tri | 1544 | *vul | 1734 | Rumex acetosa | 2022 | Symph alb |
| 2241 | Aescu hip | 251 | Brass nap | 2547 | *off | 763 | Eupat can | 1016 | tet | 1291 | Mercu per | 1546 | Polys acu | 1735 | acetosell | 2024 | Symph off |
| 21 | Aethu cyn | 251.1 | nap ole | 1592 | Comar pal | 772 | Eupho hel | 1020 | Hypoc rad | 1296 | Miliu eff | 1548 | set | 1741 | conglo | 2025 | x upl |
| 22 | Agrim eup | 256 | Briza med | 540 | Coniu mac | 777 | peplus | 1023 | Ilex aqu | 4328 | Mimul gut | 1549 | Popul alb | 1742 | crisp | 2029 | Syrin vul |
| 35.2 | Agros can | 272 | Bromo ram | 541 | Conop maj | 798 | Euphr nem | 1026 | Impat gla | 1305 | Moehr tri | 4313 | nig | 1748 | obt | 2032 | Tamus com |
| 35 | *can | 269 | Bromu hor | 544 | Convo arv | 2243 | *agg | 1038 | Iris pse | 1307 | Molin cae | 1553 | nig 'Ita' | 1753 | san | 503 | Tanac par |
| 40 | cap | 277 | Buddl dav | 548 | Cornu san | 810 | Fagus syl | 1047 | Isole set | 1312 | Monti fon | 1555 | tre | 1766 | Sagin nod | 2033 | vul |
| 39 | sto | 2249 | Calli*agg | 557 | Coryl ave | 1527 | Fallo con | 1050 | Juncu acutif | 1315 | Mycel mur | 1550 | x cana | 1767 | pro | 2034 | Tarax*agg |
| 35.1 | vin | 307 | *sta | 560 | Coton hor | 1528 | jap | 1054 | art | 1317 | Myoso arv | 1551 | x cane | 1784 | Salix alb | 2039 | Taxus bac |
| 42 | Aira pra | 309 | Callu vul | 563 | sim | 822.2 | Festu fil | 1057.2 | buf | 1321 | disco | 1563 | Potam cri | 1787 | aur | 2046 | Teucr scoro |
| 46 | Ajuga rep | 310 | Calth pal | 4526 | *agg | 822 | ovi | 1057 | *buf | 1319 | lax | 1570 | nat | 1788 | cap | 2058 | Thlas arv |
| 47 | Alche acu | 311 | Calys sep | 2423 | Crass hel | 821 | *ovi | 1058 | bul | 1322 | sco | 1574 | pec | 1789 | cin | 2060 | Thymu pol |
| 4480 | fil | 311.2 | sep sep | 569 | Crata mon | 824 | *rub | 1063 | con | 1325 | syl | 1576 | pol | 1786 | cin ole | 2063 | Tilia cor |
| 57 | fil ves | 313 | sil | 572 | Crepi cap | 1649 | Ficar ver | 1067 | eff | 1331 | Myrio spi | 1584 | Poten ans | 1793 | fra | 2064 | pla |
| 51 | glab | 316 | Campa lat | 576 | pal | 1649.2 | ver fert | 1070 | inf | 1333 | Myrrh odo | 1588 | ere | 1799 | pen | 2065 | x euro |
| 2255 | mol | 322 | rot | 580 | Croco x cro | 833 | Filip ulm | 1075 | squ | 2614 | Narci*agg | 1594 | rep | 1800 | phy | 2069 | Toril jap |
| 58 | *vul | 325 | Capse bur | 875 | Cruci lae | 838 | Fraga ves | 1080 | Junip com | 1343 | pse | 1596 | ste | 1801 | pur | 2074 | Trago pra |
| 60 | xan | 327 | Carda ama | 592 | Cymba mur | 841 | Fraxi exc | 1084 | Knaut arv | 1344 | Nardu str | 5442 | Poter san | 1805 | vim | 2080 | Trifo cam |
| 63 | Alism pla | 328 | fle | 597 | Cynos cri | 854 | Fumar off | 1087 | Koele mac | 1348 | Nastu*off | 1599 | san san | 1815 | Sambu nig | 2081 | dub |
| 64 | Allia pet | 329 | hir | 603 | Cysto fra | 860 | Galan niv | 1091 | Labur ana | 1347 | off | 1605 | Primu ver | 1818 | Sangu off | 5459 | hyb |
| 75 | Alliu urs | 331 | pra | 1822 | Cytis sco | 869 | Galeo tet | 1095 | Lactu vir | 1173 | Neott ova | 1607 | vul | 1819 | Sanic eur | 2087 | med |
| 76 | vin | 335 | Cardu cri | 607 | Dacty glo | 868 | *tet | 862 | Lamia gal | 1358 | Nymph alb | 1610 | Prune vul | 1846 | Scabi col | 2091 | pra |
| 2406 | Alnus cor | 337 | nut | 608 | Dacty fuc | 879 | Galiu alb | 2667 | gal arg | 1361 | Odont ver | 1611 | Prunu avi | 813 | Sched aru | 2092 | rep |
| 77 | glu | 341 | Carex acuti | 613 | pur | 873 | apa | 1098 | Lamiu alb | 1363 | Oenan cro | 1612 | cer..ra | 816 | gig | 2102 | Trigl pal |
| 78 | inc | 350 | bin | 2863 | x ven | 183 | odo | 1103 | pur | 1377 | Ononi rep | 1614 | dom | 823 | pra | 1241.1 | Tripl ino |
| 82 | Alope gen | 355 | car | 1915 | Danth dec | 882 | pal | 1104 | Lapsa com | 1381.2 | Ophio vul | 1615 | lau | 1851 | Schoe lac | 1241.3 | mar |
| 85 | pra | 361 | demis | 5474 | Daucu car | 878 | sax | 1105 | Larix dec | 1382 | Ophry api | 1616 | pad | 1852 | tab | 2105 | Trise fla |
| 98 | Anaca pyr | 367 | disti | 620 | car car | 887 | uli | 2302 | kae | 1387 | Orchi mas | 1617 | spi | 1129 | Scorz aut | 4367 | Triti aes |
| 99 | Anaga arv | 370 | ech | 627 | Desch ces | 888 | verum | 2303 | x mar | 2051 | Oreop lim | 556 | Pseud lut | 1865 | Scrop aur | 2106 | Troll eur |
| 105 | Anemo nem | 376 | flac | 627.1 | ces ces | 897 | Genti ama | 1112 | Lathy lin | 1393 | Origa vul | 1619 | Pterid aqu | 1867 | nod | 2109 | Tussi far |
| 109 | Angel syl | 381 | hir | 628 | fle | 907 | Geran dis | 1116 | pra | 1413 | Oxali ace | 1620 | Pucci dis | 1875 | Sedum acr | 2111 | Typha lat |
| 113 | Anisa ste | 387 | lepid | 640 | Digit pur | 909 | luc | 1126 | Lemna minor | 1426 | Papav dub | 1625 | Pulic dys | 1876 | alb | 2112 | Ulex eur |
| 121 | Antho odo | 397 | lepor | 645 | Diplo tenuif | 911 | mol | 1128 | tri | 7046 | *dub | 1635 | Querc cer | 1891 | Senec aqu | 2119 | Ulmus gla |
| 125 | Anthr syl | 393 | nig | 646 | Dipsa*ful | 914 | pra | 1130 | Leont his | 1430 | rho | 1638 | pet | 1896 | eru | 2122 | proc |
| 126 | Anthy vul | 396 | otr | 648 | Doron par | 917 | pyr | 502 | Leuca vul | 1431 | som | 1640 | rob | 1899 | jac | 2126 | Urtic dio |
| 131 | Aphan*agg | 399 | pal | 662.1 | Dryop aff | 918 | rob | 2621 | x sup | 1440 | Pasti sat | 1639 | x ros | 1902 | squ | 2128 | ure |
| 137 | Apium nod | 400 | panicea | 662 | *aff | 920 | san | 2250 | Ligus ova | 1443 | Penta sem | 1642 | Ranun acr | 1903 | syl | 2136 | Vacci myr |
| 141 | Aquil vul | 401 | panicula | 662.2 | bor | 921 | syl | 1144 | vul | 1521 | Persi amph | 1643.1 | aqu | 1904 | vis | 2139 | Valer dio |
| 142 | Arabid tha | 404 | pen | 662.3 | cam | 924 | Geum riv | 1160 | Linar pur | 1525 | bis | 1645 | aur | 1905 | vul | 2140 | off |
| 150 | Arcti*min | 405 | pil | 666 | car | 925 | urb | 1164 | vul | 1537 | mac | 1647 | bul | 1908 | Sesle cae | 2157 | Verba tha |
| 153 | nem | 408 | pul | 661 | dil | 923 | x int | 1169 | Linum cat | 1447 | Petas hyb | 1651 | fla | 1916 | Silau sil | 2165 | Veron arv |
| 162 | Arena ser | 412 | rem | 665 | fil | 931 | Glech hed | 1182 | Loliu mul | 1454 | Phala aru | 1655 | lin | 1259 | Silen dio | 2166 | bec |
| 166 | Armer mar | 413 | rip | 670 | Echiu vul | 933 | Glyce flu | 1183 | per | 1461 | Phleu ber | 1660 | repens | 1210 | flo | 2168 | cha |
| 167 | Armor rus | 414 | ros | 675 | Eleoc pal | 934 | max | 1188 | Lonic per | 1463 | pra | 1663 | sce | 1258 | lat | 2169 | fil |
| 169 | Arrhe ela | 421 | syl | 681 | Elode can | 936 | not | 1191 | Lotus cor | 2247 | *pra | 5439 | subg. Bat | 4578 | vul | 2171 | hed |
| 175 | Artem vul | 7117 | *vir | 7006 | Elymu can | 941 | Gnaph uli | 1194 | ped | 1465 | Phrag aus | 1672 | Resed lutea | 1260 | x ham | 2172 | mon |
| 176 | Arum mac | 427 | Carli vul | 33 | Elytr rep | 948.1 | Gymna con | 1195 | Lunar ann | 1470 | Picea abi | 1673 | luteola | 1933 | Sinap arv | 2173 | off |
| 185.1 | Asple adi | 428 | Carpi bet | 684 | Empet nig | 948 | *con | 1201 | Luzul cam | 2401 | sit | 1678 | Rhina min | 1938 | Sisym off | 2175 | pers |
| 192 | rut | 432 | Casta sat | 699 | Epilo bru | 2050 | Gymno dry | 1204 | mul | 4516 | Pilos aur | 1687 | Rhodo pon | 1947 | Solan dul | 2179 | scu |
| 1466 | sco | 435 | Catap rig | 688 | cil | 952 | Heder hel | 1207 | pil | 976 | off | 1694 | Ribes nig | 2251 | Solid can | 2180 | ser |
| 194 | tri | 2422 | Centa mon | 692 | hir | 952.1 | hel hel | 1209 | syl | 1476 | Pimpi sax | 1696 | rub | 2434 | gig | 2180.2 | ser ser |
| 194.1 | tri qua | 444 | nig | 695 | mon | 955 | Helia num | 1221 | Lysim nem | 1481 | Pingu vul | 2433 | san | 1951 | vir | 2185 | Vibur opu |
| 4494 | Aster novi | 446 | sca | 696 | obs | 966 | Herac man | 1223 | pun | 1482 | Pinus nig | 1697 | uva | 1952 | Sonch arv | 2189 | Vicia cra |
| 204 | tri | 5486 | Centa ery | 697 | pal | 968 | sph | 1225 | vul | 1484 | syl | 1704 | Rorip syl | 1953 | asp | 2191 | hir |
| 211 | Athyr fil | 455 | Centr rub | 698 | par | 975 | Hespe mat | 1227 | Lythr sal | 1485 | Plant cor | 7533 | Rosa cae | 1954 | ole | 2516 | sat |
| 217 | Atrip lit | 467 | Ceras fon | 705 | Epipa hel | 2560 | Hiera*agg | 1230.1 | Malus pum | 1487 | lan | 1710 | cae cae | 1958 | Sorbu ari | 2649 | sat seg |
| 218 | pat | 466 | glo | 712 | Equis arv | 3146 | sab | 1230 | *syl | 1488 | maj | 2554 | cae vos | 1957 | *ari | 2198 | sep |
| 214 | pro | 470 | tom | 713 | flu | 3240 | vul | 1232 | Malva mos | 1489 | mar | 1709 | can | 1960 | auc | 2205 | Vinca min |
| 220 | Avena fat | 555 | Cerat cla | 714 | hye | 980 | Hippo rha | 1236 | syl | 1490 | med | 1708 | *can | 1966 | int | 2206 | Viola arv |
| 961 | Avenu pra | 476 | Chaer tem | 717 | pal | 981 | Hippu vul | 1239 | Matri cha | 1495 | Poa ann | 1723 | mol | 5483 | *int | 2210 | hir |
| 962 | pub | 477 | Chame ang | 720 | syl | 983 | Holcu lan | 1242 | dis | 1506.5 | hum | 1722 | *mol | 1981 | Sparg ere | 2212 | lut |
| 225 | Ballo nig | 480 | Cheli maj | 721 | tel | 984 | mol | 1246 | Mecon cam | 1504 | nem | 1716 | rug | 1990 | Sperg mar | 2214 | odo |
| 229 | Barba vul | 482 | Cheno alb | 726 | Erica cin | 992 | Horde mur | 1250 | Medic lup | 1506 | *pra | 1718 | she | 2003 | Stach pal | 2215 | pal |
| 231 | Belli per | 484 | bon | 731 | tet | 686 | Hyaci his | 7129 | sat | 1506.4 | pra | 1719 | spi | 2005 | syl | 2218 | riv |

Please cross through the species names only. */agg refers to aggregate species encompassing other taxa. Version 3b, 2011
