# Supplementary material for: A benchmark survey of the common plants of South Northumberland and Durham, United Kingdom
Source: Biodivers Data J. 2015 Dec 29;(3):e7318. doi: 10.3897/BDJ.3.e7318 (PMC4699291; doi:10.3897/BDJ.3.e7318)
Supplement: Supplementary material 4 — Recording card for South Northumberland [file biodiversity_data_journal-3-e7318-s004.pdf]

3	Acer cam
4	pla
5	pse
7	Achil mil
9	pta
19	Adoxa mos
20	Aegop pod
2241	Aescu hip
21	Aethu cyn
22	Agrim eup
35.2	Agros can
35	*can
40	cap
39	sto
35.1	vin
41	Aira car
42	pra
46	Ajuga rep
4480	Alche fil
57	fil ves
51	glab
2255	mol
58	*vul
60	xan
63	Alism pla
64	Allia pet
75	Alliu urs
77	Alnus glu
78	inc
82	Alope gen
85	pra
97	Ammop are
99	Anaga arv
103	**Andro pol**
105	Anemo nem
109	Angel syl
113	Anisa ste
121	Antho odo
125	Anthr syl
126	Anthy vul
131	Aphan*agg
132	arv
141	Aquil vul
142	Arabid tha
150	Arcti*min
153	nem
162	Arena ser
162.2	ser ser
166	**Armer mar**
167	Armor rus
169	Arrhe ela
175	Artem vul
176	Arum mac
185.1	Asple adi
192	rut
1466	sco
194	tri
194.1	tri qua
204	Aster tri
211	Athyr fil
217	Atrip lit
218	pat
214	pro
961	Avenu pra
962	pub
225	Ballo nig
229	Barba vul
231	Belli per
237	Beton off
239	Betul pen

240	pub
243	Black per
244	Blech spi
245	**Blysm com**
250	Brach syl
251	Brass nap
256	Briza med
272	Bromo ram
269	Bromu hor
277	Buddl dav
2249	Calli*agg
307	*sta
309	Callu vul
310	Calth pal
311	Calys sep
311.2	sep sep
313	sil
316	Campa lat
322	rot
325	Capse bur
327	Carda ama
328	fle
329	hir
331	pra
335	Cardu cri
341	Carex acuti
344	are
350	bin
359	cur
355	car
361	demis
365	dio
367	disti
370	ech
376	flac
381	hir
382	hos
385	lae
387	lepid
397	lepor
393	nig
396	otr
399	pal
400	panicea
401	panicula
404	pen
405	pil
408	pul
412	rem
413	rip
414	ros
421	syl
7117	*vir
428	Carpi bet
432	Casta sat
444	Centa nig
446	**sca**
5486	Centa ery
455	Centr rub
467	Ceras fon
466	glo
469	sem
470	tom
555	Cerat cla
476	Chaer tem
477	Chame ang
480	Cheli maj
482	Cheno alb
484	**bon**
505	Chrys alt

506	opp
513	Circa lut
515	Cirsi arv
518	het
520	pal
522	vul
524	Clayt sib
533	Cochl dan
2547	*off
1592	Comar pal
540	Coniu mac
541	Conop maj
544	Convo arv
548	Cornu san
557	Coryl ave
4526	Coton*agg
569	Crata mon
572	Crepi cap
574	**mol**
576	pal
580	Croco x cro
875	Cruci lae
586	Crypt cri
592	Cymba mur
597	Cynos cri
603	Cysto fra
1822	Cytis sco
1822.2	sco sco
607	Dacty glo
608	Dacty fuc
609	inc
610	mac
613	pur
2863	x ven
1915	Danth dec
5474	Daucu car
627	Desch ces
627.1	ces ces
628	fle
640	Digit pur
645	Diplo tenuif
646	Dipsa*ful
648	Doron par
657	Drose rot
662.1	Dryop aff
662	*aff
662.2	bor
666	car
661	dil
665	fil
670	Echiu vul
675	Eleoc pal
677	qui
681	Elode can
7006	Elymu can
33	Elytr rep
684	Empet nig
699	Epilo bru
688	cil
692	hir
695	mon
696	obs
697	pal
698	par
990298	Epipa dun
705	hel
712	Equis arv
713	flu
714	**hye**
717	pal

720	syl
721	tel
726	Erica cin
731	tet
738	Erinu alp
740	Eriop ang
743	**lat**
744	vag
745	Erodi*cic
4343	Eroph gla
4342	ver
753	*ver
772	**Eupho hel**
777	peplus
7310	Euphr arc
785	arc bor
788	con
798	nem
2243	*agg
810	Fagus syl
1527	**Fallo con**
1528	jap
822.2	Festu fil
822	ovi
821	*ovi
824	*rub
1649	Ficar ver
1649.2	ver fert
833	Filip ulm
838	Fraga ves
841	Fraxi exc
854	Fumar off
860	Galan niv
869	Galeo tet
868	*tet
879	Galiu alb
873	apa
880	mol mol
183	odo
882	pal
882.2	pal pal
878	sax
887	uli
888	verum
893	Genis tin
897	Genti ama
907	Geran dis
909	luc
911	mol
914	pra
918	rob
920	san
921	syl
924	Geum riv
925	urb
923	x int
931	Glech hed
932	Glyce dec
933	flu
936	not
941	Gnaph uli
948.1	Gymna con
948	*con
2050	Gymno dry
952	Heder hel
952.1	hel hel
955	Helia num
968	Herac sph
975	Hespe mat
2560	Hiera*agg

3146	sab
981	**Hippu vul**
983	Holcu lan
984	mol
992	Horde mur
1217	Huper sel
686	Hyaci his
687	non
2617	x mas
999	Hydro vul
1010	Hyper hirsut
1014	per
1015	pul
1016	tet
1005	x des
1020	Hypoc rad
1023	Ilex aqu
1026	Impat gla
1038	Iris pse
1047	Isole set
1050	Juncu acutif
1054	art
1057.2	buf
1057	*buf
1058	bul
1063	con
1067	eff
1070	inf
1075	squ
1080	Junip com
1084	Knaut arv
1087	**Koele mac**
1091	Labur ana
2667	Lamia gal arg
1098	Lamiu alb
1103	pur
1104	Lapsa com
1104.1	com com
1105	Larix dec
2303	x mar
1107	Lathr squ
1112	Lathy lin
1116	pra
1126	Lemna minor
1128	tri
1130	Leont his
333	Lepid dra
502	Leuca vul
2621	x sup
2250	Ligus ova
1144	vul
1160	Linar pur
1164	vul
1169	Linum cat
1183	Loliu per
1188	Lonic per
1191	Lotus cor
1194	ped
1195	Lunar ann
1201	Luzul cam
1204	mul
1207	pil
1209	syl
1215	**Lycop cla**
1221	Lysim nem
1223	pun
1225	vul
1227	Lythr sal
1230.1	Malus pum
1230	*syl

1232	Malva mos
1236	syl
1242	Matri dis
1246	Mecon cam
1250	Medic lup
1256	Melam pra
1263	Melic uni
1265	Melil alt
1272	Menth aqu
1285	spi
1289	Menya tri
1291	Mercu per
1296	Miliu eff
1298	Mimul*agg
4328	gut
1305	Moehr tri
1307	Molin cae
1312	Monti fon
1315	Mycel mur
1317	Myoso arv
1321	disco
1319	lax
1320	ram
1322	sco
1323	sec
1325	syl
1330	Myrio alt
1331	spi
1333	Myrrh odo
2614	Narci*agg
1344	Nardu str
1345	Narth oss
1348	Nastu*off
1347	off
1172	Neott cor
1173	ova
2057	**Nocca cae**
1361	Odont ver
1363	Oenan cro
1377	Ononi rep
1381.2	Ophio vul
1387	Orchi mas
2051	Oreop lim
1413	Oxali ace
1426	Papav dub
7046	*dub
1430	rho
1431	som
1437	**Parna pal**
1440	Pasti sat
1441	Pedic pal
1442	syl
1443	Penta sem
1521	Persi amph
1525	bis
1537	mac
1447	Petas hyb
1454	Phala aru
2053	Phego con
1461	Phleu ber
1463	pra
2247	*pra
1465	Phrag aus
1470	Picea abi
2401	sit
976	Pilos off
1476	Pimpi sax
1481	Pingu vul
2402	Pinus con
1484	syl

1487	Plant lan
1488	maj
1489	mar
1490	**med**
1495	Poa ann
1506.5	hum
1504	nem
1506	*pra
1506.4	pra
1507	tri
1514	Polyg ser
1515	vul
1520	Polyg ar..rum
1523	avi
1522	*avi
1544.2	Polyp vul
1544	*vul
1546	Polys acu
1549	Popul alb
1555	tre
1563	Potam cri
1570	nat
1576	pol
1584	Poten ans
1588	ere
1594	rep
1596	ste
5442	Poter san
1599	san san
1605	Primu ver
1607	vul
1610	Prune vul
1611	Prunu avi
1614	dom
1615	lau
1616	pad
1617	spi
556	Pseud lut
1619	Pterid aqu
1620	Pucci dis
1625	Pulic dys
1638	Querc pet
1640	rob
1642	Ranun acr
1643.1	aqu
1645	aur
1647	bul
1651	fla
1653	hed
1655	lin
1654	omi
1660	repens
1663	sce
5439	subg. Bat
1672	Resed lutea
1673	luteola
1678	Rhina min
1679	min min
1687	Rhodo pon
1694	Ribes nig
1696	rub
2433	san
1695	**spi**
1697	uva
1703	Rorip pal
7533	Rosa cae
1709	can
1708	*can
1723	mol
1722	*mol

1716	rug
1718	she
1726	**Rubus caesi**
1727	cha
1728	*fru
1729	ida
1730	**sax**
1734	Rumex acetosa
1735	acetosell
1736	ace ace
1741	conglo
1742	crisp
1746	lon
1748	obt
1753	san
2559	Sagin ape
1766	nod
1767	pro
1784	Salix alb
1787	aur
1788	cap
1789	cin
1786	cin ole
1793	fra
1799	pen
1800	phy
1801	pur
1802	rep
1805	vim
1815	Sambu nig
1818	Sangu off
1819	Sanic eur
1830	**Saxif gra**
1846	Scabi col
813	Sched aru
816	gig
823	pra
1851	Schoe lac
1861	Scirp syl
1129	Scorz aut
1865	Scrop aur
1867	nod
1872	Scute gal
1875	Sedum acr
1876	alb
1888	Selag sel
1891	Senec aqu
1899	jac
1902	squ
1903	syl
1904	vis
1905	vul
1916	Silau sil
1259	Silen dio
1210	flo
1258	lat
4578	vul
1933	Sinap arv
1938	Sisym off
1947	Solan dul
1951	Solid vir
1952	Sonch arv
1953	asp
1954	ole
1958	Sorbu ari
1957	*ari
1960	auc
1966	int
1983	Sparg eme
1981	ere

1990	Sperg mar
1992	rub
2003	Stach pal
2005	syl
2007	Stell als
2009	gra
2010	hol
2012	med
2014	nem
2021	Succi pra
2022	Symph alb
2024	Symph off
2025	x upl
2029	Syrin vul
503	Tanac par
2033	vul
2034	Tarax*agg
2039	Taxus bac
2046	Teucr scoro
2049	Thali min
2058	Thlas arv
2060	Thymu pol
2065	Tilia x euro
2069	Toril jap
2074	Trago pra
1858.2	Trich ger
1858.3	ces foe
2076	Trien eur
2080	Trifo cam
2081	dub
2087	med
2091	pra
2092	rep
2102	Trigl pal
1241.1	Tripl ino
1241.3	mar
2105	Trise fla
2106	Troll eur
2109	Tussi far
2111	Typha lat
2112	Ulex eur
2119	Ulmus gla
2126	Urtic dio
2136	Vacci myr
1419	oxy
2138	vit
2139	Valer dio
2140	off
2157	Verba tha
2165	Veron arv
2166	bec
2168	cha
2169	fil
2171	hed
2172	mon
2173	off
2175	pers
2179	scu
2180	ser
2180.2	ser ser
2185	Vibur opu
2189	Vicia cra
2191	hir
2516	sat
2198	sep
2206	Viola arv
2212	**lut**
2215	pal
2218	riv
2220	**tri**

Please cross through the species names only. */agg refers to aggregate species encompassing other taxa. Any taxa in bold are of Conservation Concern and a detailed record should be made. Version 3b, 2011
